# Is there a problem with how we select patients for therapeutic hypothermia?

**DOI:** 10.1038/s41390-024-03313-1

**Published:** 2024-06-07

**Authors:** Eleanor R. Gunn, Malcolm R. Battin, Alistair J. Gunn

**Affiliations:** 1 Newborn Services, Te Whatu Ora Counties Manukau, Auckland, New Zealand; 2 Newborn Services, Auckland City Hospital, Auckland, New Zealand; 3 Department of Physiology, The University of Auckland, Auckland, New Zealand

It has been over a decade since therapeutic hypothermia (TH) was shown in multiple large randomized controlled trials to improve the survival without disability after moderate-to-severe-hypoxic ischemic encephalopathy (HIE)^1^ and became part of standard care.^2^ It remains the only proven treatment for HIE. Although there are multiple causes of neonatal encephalopathy, only HIE has been shown to benefit from TH.^3^ The criteria used in the original trials were developed by multiple centers working separately. Unsurprisingly, this resulted in minor protocol differences. Overall, all entry criteria aimed to select infants with a relatively high risk of adverse outcome (i.e. event rate, which could be modified by TH) and therefore required the combination of evidence for a recent hypoxic ischemic insult (such as metabolic acidosis, need for resuscitation) plus moderate to severe encephalopathy on examination. Clearly, strict entry criteria are needed in a trial context where a homogenous study population and power to show statistically significant benefit are important considerations. However, using such narrow criteria in routine clinical practice may come at the cost of excluding some infants who might benefit.^4,5^

In this issue, Proietti et al. highlight variation in guidelines and consensus statements around the world in eligibility criteria for therapeutic hypothermia,^6^ including the assessment of HIE. The authors suggest that this variation risks unequal provision of care to vulnerable infants based on their place of birth, and so could lead to some infants who might benefit missing out on life-changing therapy. It is reasonable to consider that exclusion from clinical guidelines does not necessarily mean that an infant will not benefit from cooling, particularly given the now proven relative safety of TH. For example, although hypothermia should optimally be started as early as possible within 6 h after birth,^7^ there is trial evidence of a probable, albeit very small potential benefit even if therapeutic hypothermia is started after 6 h.^8^ The individual clinician will naturally take this into account.

Although variation between guidelines may in principle lead to unequal management for infants with HIE, despite relatively small differences in their entry criteria, the outcomes of the randomized controlled trials in high income countries were strikingly homogenous in meta-analysis,^1^ suggesting that minor differences in recruitment criteria have little effect on success in recruiting infants who could benefit. A significant proportion of the documents examined for variation in the paper in this issue^6^ are Consensus statements, which acknowledge such uncertainty in addition to drawing on local system knowledge. Moreover, some encourage discussion with experts at tertiary centers when infants may lie outside rigid criteria but might still benefit from TH.

It has been recognized that increasing access to TH based solely on expanded criteria and clinical judgment could be associated with unwanted consequences including overtreatment, increased use of TH for the wrong diagnosis or iatrogenic treatment problems.^9^ However, translation to routine practice naturally necessitates criteria that are adapted to fit real world practicalities. One important driver for such adaptation is recognition of widespread incomplete adherence to current criteria as evidenced by the therapeutic drift of TH in mild HIE.

Resource limitations and local experience will, at least in part, affect the practicality of some assessment criteria. The use of aEEG and EEG has increased since the introduction of TH and provide important information on the diagnosis and severity of encephalopathy as well as seizure identification.^10^ However, some neonatal units may not yet have timely access to this technology, particularly as some infants will be outborn and, optimally, monitoring needs to be started early in the postnatal course. In addition, there may be barriers linked to cost or lack of local expertize. Fortunately, automated interpretation is progressively improving, to the point that it may soon be viable for general use in the early postnatal period.^11^

Clinical assessment of the severity of encephalopathy therefore remains the foundation of assessment of probable HIE. The original Sarnat classification/staging has been adapted to provide more finely graded NICHD and SIBEN scores. In head-to-head comparison, the systems show good concordance, and both numerical scores were superior to Sarnat in predicting MR injury.^12^ The reader should note that SIBEN defined more cases as moderate, and fewer as mild, than NICHD.

In conclusion, the real clinical challenge in most countries is not that there are differences in the fine detail of their current guidelines, but rather that hypothermia is increasingly being used well outside guidelines, particularly for much milder HIE than tested in the original trials. We know that mild HIE identified in the first 6 h of life can still be associated with significant risk of cerebral palsy and lower cognitive scores.^4,5^ Thus, therapeutic hypothermia for “mild” HIE has been identified as one of 5 top priorities for research by stakeholders.^13^ For now, it is arguable that rather than relitigating old criteria we should focus on clarifying whether and for whom hypothermia is beneficial. A quantitative approach in which all elements of encephalopathy are graded and if possible combined with early EEG monitoring may more effectively identify infants with mild HIE who are at risk of adverse outcomes.^12,14^ Although implementing this type of approach would require widespread multi-national ongoing investment across both service providers and special interest groups, we speculate that adoption of such an approach would improve the consistency of documentation around encephalopathy severity. In turn, this may improve consistency of recruitment and allow better targeting of therapy in future large trials.
